# Conservation agriculture based integrated crop management sustains productivity and economic profitability along with soil properties of the maize-wheat rotation

**DOI:** 10.1038/s41598-022-05962-w

**Published:** 2022-02-04

**Authors:** Vijay Pooniya, R. R. Zhiipao, Niraj Biswakarma, Dinesh Kumar, Y. S. Shivay, Subhash Babu, Kajal Das, A. K. Choudhary, Karivaradharajan Swarnalakshmi, R. D. Jat, R. L. Choudhary, Hardev Ram, Mukesh K. Khokhar, Ganapati Mukri, K. K. Lakhena, M. M. Puniya, Rajkumar Jat, L. Muralikrishnan, A. K. Singh, Achal Lama

**Affiliations:** 1 ICAR-Indian Agricultural Research Institute (IARI), New Delhi, 110 012 India; 2 ICAR-Central Research Institute for Jute and Allied Fibers, Barrackpore, West Bengal 700120 India; 3 ICAR-Central Potato Research Institute (CPRI), Shimla, 171001 India; 4 Chaudhary Charan Singh Haryana Agricultural University (CCSHAU), Hisar, Haryana 125004 India; 5 ICAR-Directorate of Rapeseed-Mustard Research, Bharatpur, Rajasthan 321303 India; 6 ICAR-National Dairy Research Institute, Karnal, Haryana 132001 India; 7 ICAR - National Centre for Integrated Pest Management, New Delhi, 110 012 India; 8 Agriculture University, Jodhpur, Mandor, Jodhpur, 342304 India; 9 Borlaug Institute for South Asia (BISA), Samastipur, Bihar, 848125 India; 10 ICAR-Indian Institute of Maize Research (IIMR), PAU Campus, Ludhiana, Punjab 141 004 India; 11 ICAR-Indian Agricultural Statistics Research Institute (IASRI), New Delhi, 110 012 India

**Keywords:** Climate-change impacts, Ecosystem ecology, Environmental impact

## Abstract

Field experiments were conducted to evaluate eight different integrated crop management (ICM) modules for 5 years in a maize-wheat rotation (M_WR_); wherein, ICM_1&2_-ˈbusiness-as-usualˈ (conventional flatbed maize and wheat, ICM_3&4_-conventional raised bed (CT_RB_) maize and wheat without residues, ICM_5&6_-conservation agriculture (CA)-based zero-till (ZT) flatbed maize and wheat with the residues, and ICM_7&8-_ CA-based ZT raised bed maize and wheat with the residues. Results indicated that the ICM_7&8_ produced significantly (p < 0.05) the highest maize grain yield (5 years av.) which was 7.8–21.3% greater than the ICM_1-6_. However, across years, the ICM_5-8_ gave a statistically similar wheat grain yield and was 8.4–11.5% greater than the ICM_1-4_. Similarly, the CA-based residue retained ICM_5-8_ modules had given 9.5–14.3% (5 years av.) greater system yields in terms of maize grain equivalents (M_GEY_) over the residue removed CT-based ICM_1&4_. System water productivity (S_WP_) was the highest with ICM_5-8_, being 10.3–17.8% higher than the ICM_1-4_. Nevertheless, the highest water use (T_WU_) was recorded in the CT flatbed (ICM_1&2_), ~ 7% more than the raised bed and ZT planted crops with or without the residues (ICM_4-8_). Furthermore, the ICM_1-4_ had produced 9.54% greater variable production costs compared to the ICM_5-8_, whereas, the ICM_5-8_ gave 24.3–27.4% additional returns than the ICM_1-4_. Also, different ICM modules caused significant (p < 0.05) impacts on the soil properties, such as organic carbon (S_OC_), microbial biomass carbon (S_MBC_), dehydrogenase (S_DH_), alkaline phosphatase (S_AP_), and urease (U_RE_) activities. In 0.0–0.15 m soil profile, residue retained CA-based (ICM_5-8_) modules registered a 7.1–14.3% greater S_OC_ and 10.2–17.3% S_MBC_ than the ICM_1-4_. The sustainable yield index (S_YI_) of M_WR_ was 13.4–18.6% greater under the ICM_7&8_ compared to the ICM_1-4._ Hence, this study concludes that the adoption of the CA-based residue retained ICMs in the M_WR_ could sustain the crop yields, enhance farm profits, save water and improve soil properties of the north-western plans of India.

Globally, maize (*Zea mays* L.) is the 3rd most important cereal, and across ecologies, being grown in ~ 155 nations; *called ˈQueen of cereals*ˈ (maize), the back bone of American food or a miracle crop. The United State produced ~ 31% of the maize grains, subsequently China (24%), Brazil (8%) and India (2.2%)^1,2^. In India, the maize-wheat rotation (M_WR_) is the 5th leading cropping rotation, occupying ∼2 million ha in the Indo-Gangetic Plains (I_GPs_), the heart land of the rice–wheat rotation (R_WR_)^3^. The relatively greater yields of the R_WR_ in the upper I_GPs_ materialized at the costs of the over utilization of the natural resources^4,5^, which caused nutrient imbalances, greater energy use and increased labour demands, weed shift/resistance and more G_HGs_ emissions^6,7^. Further, rice residue burning is one of the realised threats of the R_WR_ sustainability, which resulted in the extensive impacts on the losses of soil organic matter (S_OM_) and nutrients, reduced biodiversity, lowered water and energy efficiency, and of course the declined air quality. In India’s capital and other adjoining north Indian cities, the residue burning reduces air quality, with severe impacts on human and animal health^8,9^. Hence, these ruinous factors have given impetus to pursue alternative crops/rotations or to follow the integrated sustainable strategies in the line of UN Sustainable Development Goals, i.e., more environmentally sound and efficient utilizer of resources^10–12^.

The maize adaptability to diverse agro-ecologies or across seasons is unmatched to any other crops. It can be a feasible alternative to the rice in R_WR_, and a potential driver for the crop diversification^13,14^. In India, it covers ~ 9.5 million hectares with 24.5 million tonnes annual production, and 3rd most important food crop next to rice and wheat^2^. It is consumed in the form of grains, green cobs, sweet corn, baby corn and popcorn, besides its use as animal feed, fodder and raw material for the industrial products such as food (25%), animal feed (12%), poultry feed (49%), starch (12%), brewery and seed^15^. The intensive tillage with crop establishment accounts ~ 25% of the total production cost, leading to the reduced net income^16^. Here, the major challenge is to develop the alternative production system that should be climate and resource resilient, and can help to sustain the crop yields in the long-run^17^. Recently, the CA-based crop management, such as no-till or zero-till and bed plantings with residue retention and judicious crop rotations, is gaining more attention with the rising concerns pertaining to the over degradation of the natural resources, to offset the production cost^18^. Both the crops (maize, wheat) could be well fitted, and may prove input responsive in the CA-based practices^19,20^. A great potential exists to raise the yields and sustainability of the maize-wheat rotation (M_WR_) further by combining the CA-based production with certain integrated crop management (ICM) practices. Thus, need was felt to find out the best combinations of the ICM practices to accomplish the sustainability of the M_WR_. It is reported that these ICM practices can help in the initial crop establishment with greater input efficiency, and open up avenues for CA-based ICMs which could further help in the timely seeding of the both crops, hence may lead to the sustained yields without compromising the degradation of the natural resources.

Recently, the Food and Agriculture Organisation (FAO) has suggested that the ICM is of much significance and relevance than the individual agronomic management approach. The ICM is fundamentally based on the understanding of the interactions between the biology, environment and the land management systems apart from conserving the natural resources and producing the food on an economically viable and sustainable platform^21^. Adoption of the ICM practices significantly improved the crop yields to the tune of 20–30% in India^22^, and 13.5% in China^23^ over the farmers’ practice, while minimizing the production costs simultaneously^24,25^. In R_WR_, a recent long-term study showed the superiority of the ICM-based modules, with 10–13% greater system yields, saved 8–12% irrigation water, and gave 19–22% additional economic returns over the CT-based modules^5^.

Therefore, the integration of the ICM practices along with the CA-background needs to be developed in a holistic manner so as to achieve the long-term sustainability and profitability of the M_WR_. With this hypothesis, we have evaluated the different ICM modules for five years in a M_WR_ of the north-western India, chiefly aimed to improve the crop and water productivity, economic profitability, sustainability and soil biological properties.

## Results

### Five years’ trends and pooled maize grain and stover yields

**Figure 1:**
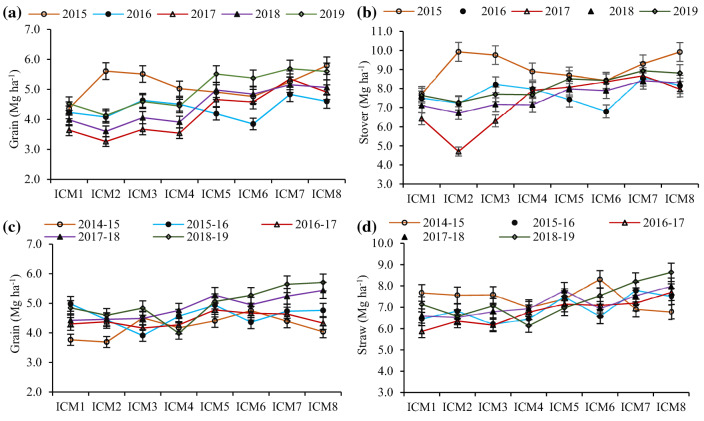
Five years' maize grain and stover (**a**,**b**); wheat grain and straw (**c**,**d**) yield trend under different ICM modules in maize-wheat rotation. The vertical bars indicate LSD at p = 0.05.

During the initial year, the maize grain yield did not differ significantly among the ICM modules, although the highest yield was recorded under the ICM_8_. Nevertheless, from the second year onwards, the different ICM modules had the significant (p < 0.05) impacts on the maize grain yield (Fig. 1a). The ICM_7_ consistently produced the highest yield across the years, which was closely followed by the ICM_8_. Similarly, the highest stover yield across the years was recorded with ICM_7_, except first year (Fig. 1b). The highest pooled grain (5.2 Mg ha^−1^) and stover (8.7 Mg ha^−1^) yields were recorded with the ICM_7_, being close to the ICM_3-6&8_. On an average, the ICM_7&8_ had produced 5.9–21% and 5.8–18.4% greater grain and stover yields, respectively, over the ICM_1-6_ (Table 1).

**Table 1:** Five years’ pooled grain and stover / straw (Mg ha^−1^) (± S.D.) yields of maize-wheat rotation under different ICM modules. Means followed by a similar lowercase letters within a column are not significantly different at p < 0.05 according to Tukey’s HSD test.

Treatment	Maize		Wheat	
	Grain	Stover	Grain	Straw
ICM_1_	4.1^b^ ± 0.33	7.2^b^ ± 0.52	4.4^a^ ± 0.16	6.7^a^ ± 0.20
ICM_2_	4.1^b^ ± 0.11	7.1^b^ ± 0.40	4.3^a^ ± 0.23	6.7^a^ ± 0.39
ICM_3_	4.4^ab^ ± 0.27	7.8^ab^ ± 0.21	4.3^a^ ± 0.20	6.7^a^ ± 0.39
ICM_4_	4.2^ab^ ± 0.11	7.9^ab^ ± 0.26	4.3^a^ ± 0.30	6.6^a^ ± 0.50
ICM_5_	4.8^ab^ ± 0.35	8.1^ab^ ± 0.45	4.8^a^ ± 0.19	7.3^a^ ± 0.09
ICM_6_	4.6^ab^ ± 0.22	7.9^ab^ ± 0.43	4.8^a^ ± 0.35	7.2^a^ ± 0.57
ICM_7_	5.2^a^ ± 0.15	8.7^a^ ± 0.39	4.9^a^ ± 0.14	7.3^a^ ± 0.08
ICM_8_	5.1^a^ ± 0.19	8.6^a^ ± 0.16	4.8^a^ ± 0.11	7.7^a^ ± 0.04

^#^ICM_1&2_ = conventional flatbed maize & wheat (CT_FB_); ICM_3&4_ = conventional raised bed maize & wheat (CT_RB_); ICM_5&6_ = zero-till (ZT) flatbed maize with wheat residue at ~ 3 Mg ha^−1^ (ZT_M_ + W_R_) & ZT wheat with maize residue at ~ 5 Mg ha^−1^ (ZT_W_ + M_R_), and ICM_7&8_ = ZT raised bed maize with wheat residue (ZT_RB_ + W_R_) & ZT wheat with maize residue (ZT_RB_ + M_R_). ^#^R_DF_ = recommended fertilizers for maize / wheat 150:26.2:50 / 120:26:33 NPK kg ha^−1^; L_BFs_ = NPK liquid bio-fertilizer; A_MF_ = arbuscular mycorrhizal fungi.

### Five years’ trends and pooled wheat grain and straw yields

The different ICM modules did not impact the wheat grain yield significantly during the first three years. While, at the fourth year, the ICM_5_ had the highest yield, being significantly higher than the ICM_1-3_, and subsequently in the fifth year, it was ICM_8_ which outperformed significantly (p < 0.05) over the ICM_4_ (Fig. 1c). Similarly, the straw yield did not differ significantly among the ICM modules in the initial three years, but significantly a greater yield was registered with the ICM_8_ in the fourth and fifth years (Fig. 1d). However, the mean grain and straw yields under the ICM_5-8_ (CA-based ZT) was 8.4–11.5% and 7–14% greater than the CT-based residue removed (ICM_1-4_) modules (Table 1).

### System yields in terms of maize grain equivalents

The ICM modules had a significant impact on the maize grain equivalents (M_GEY_) across the years, except during the initial two years (2015–16 and 2016–17), wherein the ICM_7_ produced the highest yield during the 2017–18 and 2019–20, which was significantly greater than the ICM_1-4_ to the tune of 19–22% and 17–26%, respectively. While, in 2018–2019, the highest yield was recorded with the ICM_8_, which was significantly higher than the ICM_1-4_ by 16–22%. Averaged across the five years, the ICM_5-8_ had 6–15% system M_GEY_ advantage over the ICM_1-4_ (Table 2).

**Table 2:** Five years’ trend of system productivity (Mg ha^−1^) (± S.D.) in terms of maize grain equivalent yield (M_GEY_) of maize-wheat rotation under different ICM modules. Means followed by a similar lowercase letters within a column are not significantly different at p < 0.05 according to Tukey’s HSD test.

Treatment	System maize grain equivalents (M_GEY_)				
	2015–16	2016–17	2017–18	2018–19	2019–20
ICM_1_	8.5^a^ ± 0.94	9.9^a^ ± 0.68	8.7^bcd^ ± 0.25	9.3^bc^ ± 0.65	9.7^bc^ ± 0.54
ICM_2_	9.6^a^ ± 0.95	9.2^a^ ± 1.01	8.4^d^ ± 0.58	9.0^c^ ± 0.63	9.1^c^ ± 0.53
ICM_3_	10.5^a^ ± 0.21	9.1^a^ ± 1.38	8.6^ cd^ ± 0.30	9.5^bc^ ± 0.23	9.8^bc^ ± 1.53
ICM_4_	9.6^a^ ± 0.80	9.7^a^ ± 1.09	8.6^ cd^ ± 0.36	9.7^bc^ ± 0.26	8.7^c^ ± 0.90
ICM_5_	9.7^a^ ± 1.36	9.8^a^ ± 1.45	10.3^ab^ ± 1.11	11.4^a^ ± 0.62	10.9^ab^ ± 0.71
ICM_6_	10.0^a^ ± 1.95	8.8^a^ ± 1.16	10.1^abc^ ± 0.70	10.8^ab^ ± 0.55	11.0^ab^ ± 0.66
ICM_7_	10.1^a^ ± 0.66	10.2^a^ ± 0.66	10.8^a^ ± 0.83	11.5^a^ ± 0.45	11.8^a^ ± 1.15
ICM_8_	10.2^a^ ± 1.50	10.0^a^ ± 0.14	10.0^abc^ ± 0.51	11.6^a^ ± 0.64	11.7^a^ ± 1.03

^#^ICM_1&2_ = conventional flatbed maize & wheat (CT_FB_); ICM_3&4_ = conventional raised bed maize & wheat (CT_RB_); ICM_5&6_ = zero-till (ZT) flatbed maize with wheat residue at ~ 3 Mg ha^−1^ (ZT_M_ + W_R_) & ZT wheat with maize residue at ~ 5 Mg ha^−1^ (ZT_W_ + M_R_), and ICM_7&8_ = ZT raised bed maize with wheat residue (ZT_RB_ + W_R_) & ZT wheat with maize residue (ZT_RB_ + M_R_). ^#^R_DF_ = recommended fertilizers for maize / wheat 150:26.2:50 / 120:26:33 NPK kg ha^−1^; L_BFs_ = NPK liquid bio-fertilizer; A_MF_ = arbuscular mycorrhizal fungi.

### System water use and productivity

**Figure 2:**
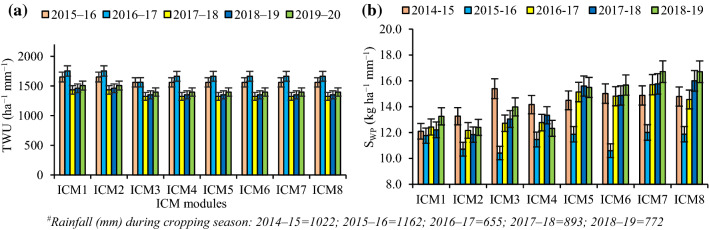
Five years' water use (**a**) and system water productivity (**b**) trend under different ICM modules in maize-wheat rotation. The vertical bars indicate LSD at p = 0.05.

The system water use (T_WU_) (irrigation + Ep) differed across the years. The highest water was consumed (1434–1753 kg ha^−1^ mm^−1^) in the ICM_1&2_, while it was relatively lesser under the ICM_3-8_ (1324–1663 kg ha^−1^ mm^−1^). On an average, the ICM_3-8_ saved 6.5% system water use compared to the ICM_1&2_ (Fig. 2a). In contrast, the highest water productivity (W_P_) was observed with the ICM_3_ (2015–16), ICM_7_ (2016–17, 2017–18), and ICM_8_ (2018–19). While, in 2019–20, the ICM_7&8_ produced the similar W_P_, but significantly higher than the ICM_1-4_. The average W_P_ under CA-based residue retained modules (ICM_5-8_) was 7.7–19.6% greater than the CT (ICM_1-4_) practices (Fig. 2b).

### System variable production costs and economic returns

**Figure 3:**
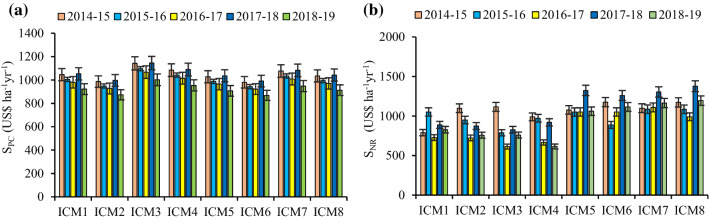
Five years' trend in system variable production costs (S_PC_) (**a**) and net returns (S_NR_) (**b**) under different ICM modules in the maize–wheat rotation. The vertical bars indicate LSD at p = 0.05.

Across the years, the variable input costs differed among the ICM modules. The highest system input cost was incurred with the ICM_3_ (US$1001–1145 ha^−1^ yr^−1^), while the least was under the ICM_6_ (US$868–991 ha^−1^ yr^−1^). On an average, the ICM_1-4_ had 9.54% greater variable production costs compared to the ICM_5-8_ (Fig. 3a). Furthermore, the ICM_7&8_ gave the highest net economic returns, resulting chiefly due to greater yields and lesser production costs incurred. The average increment in the net returns under the ICM_7&8_ was 23.6–29.5% compared to the ICM_1-4_ (Fig. 3b).

### Soil properties

**Figure 4:**
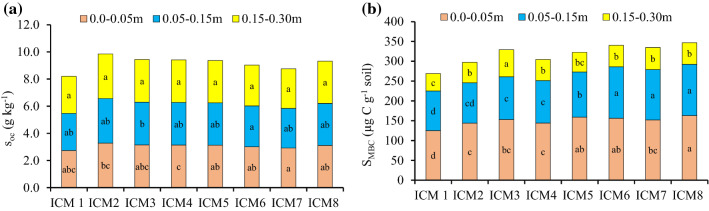
Effect of ICM modules on S_OC_ (**a**) and soil microbial biomass carbon (S_MBC_) (**b**) at different soil depths at flowering of 5th season wheat in the maize-wheat rotation. Means followed by a similar lowercase letter within a bar are not significantly different at p < 0.05 using Tukey’s HSD test.

The ICM modules had a significant impact on the variable soil properties i.e., soil organic carbon (S_oc_), microbial biomass carbon (S_MBC_), dehydrogenase activity (S_DH_), alkaline phosphatase (S_AP_) and soil urease (U_RE_) activities (Fig. 4, Table 3).

**Table 3:** Effect of different ICM modules on soil dehydrogenase activity (S_DH_), alkaline phosphatase (S_AP_) and urease (U_RE_) at the flowering of 5^th^ season wheat under M_WR_. Means followed by a similar lowercase letters within a column are not significantly different at p < 0.05 according to Tukey’s HSD test.

Treatment	S_DH_ (µg TPF g^−1^ fresh soil d^−1^)			S_AP_ (µg p-NP g^−1^ soil h^−1^)			U_RE_ (µg NH_4_-N g^−1^ soil h^−1^)		
	0.0–0.05 m	0.05–0.15 m	0.15–0.30 m	0.0–0.05 m	0.05–0.15 m	0.15–0.30 m	0.0–0.05 m	0.05–0.15 m	0.15–0.30 m
ICM_1_	58.3^c^	26.1^cd^	13.2^ab^	54.3^d^	44.6^bc^	27.1^a^	14.2^c^	11.8^c^	7.6^a^
ICM_2_	53.2^d^	22.0^d^	13.4^ab^	49.8^d^	38.9^c^	30.2^a^	15.6^bc^	12.6^bc^	6.5^a^
ICM_3_	57.4^cd^	26.5^cd^	15.7^a^	61.8^c^	44.9^bc^	32.8^a^	14.7^c^	12.3^bc^	6.1^a^
ICM_4_	62.0^bc^	28.7^bc^	13.4^ab^	52.9^d^	43.5^c^	28.9^a^	16.3^abc^	11.7^c^	8.4^a^
ICM_5_	60.9^bc^	26.5^cd^	16.4^a^	66.0^bc^	51.9^a^	30.6^a^	18.5^ab^	13.5^abc^	8.6^a^
ICM_6_	67.3^a^	29.5^abc^	15.5^a^	70.4^ab^	56.3^a^	28.9^a^	19.4^a^	14.7^ab^	7.7^a^
ICM_7_	63.3^ab^	31.9^ab^	13.8^ab^	72.0^a^	51.1^ab^	31.6^a^	18.5^ab^	13.7^abc^	7.8^a^
ICM_8_	65.2^ab^	34.7^a^	11.7^b^	73.6^a^	42.6^c^	31.4^a^	19.5^a^	16.1^a^	7.7^a^

^#^ICM_1&2_ = conventional flatbed maize & wheat (CT_FB_); ICM_3&4_ = conventional raised bed maize & wheat (CT_RB_); ICM_5&6_ = zero-till (ZT) flatbed maize with wheat residue at ~ 3 Mg ha^−1^ (ZT_M_ + W_R_) & ZT wheat with maize residue at ~ 5 Mg ha^−1^ (ZT_W_ + M_R_), and ICM_7&8_ = ZT raised bed maize with wheat residue (ZT_RB_ + W_R_) & ZT wheat with maize residue (ZT_RB_ + M_R_). ^#^R_DF_ = recommended fertilizers for maize / wheat 150:26.2:50 / 120:26:33 NPK kg ha^−1^; L_BFs_ = NPK liquid bio-fertilizer; A_MF_ = arbuscular mycorrhizal fungi.

### Soil organic carbon (S_oc_)

In the top 0.00–0.05 m soil depth, the highest S_oc_ was recorded with the ICM_7,_ which was significantly higher than the ICM_2&4_. The increment in S_oc_ under the ICM_7&8_ over the ICM_1-4_ was to the tune of 10.2–16.2%. Further, in the 0.05–0.15 m soil depth, the highest S_oc_ was recorded with the ICM_6_, wherein it was significantly more than the ICM_3,_ but statistically (p < 0.05) similar to the ICM_1,2,4,5,7&8_. While, there were no significant differences among the ICM modules, with respect to the S_oc_, in the 0.15–0.30 m soil depth (Fig. 4a).

### Soil microbial biomass carbon (S_MBC_)

The highest S_MBC_ in the 0.00–0.05 m soil depth was observed under the ICM_8_, wherein it was similar to the ICM_5&6_, but significantly higher than the ICM_1-4&7_. The ICM_8_ had 6–23% greater S_MBC_ than the ICM_1-4_. While, in the 0.05–0.15 m soil depth, the highest S_MBC_ was recorded in the ICM_6_, being significantly greater than the ICM_1-5_ to the tune of 12–22.8%, but similar to the ICM_7&8_. In contrast, at lower soil depth (0.15–0.30 m), the highest S_MBC_ was observed under the ICM_3,_ and being greater than that of the ICM_1,2&4–8_ (Fig. 4b).

### Soil dehydrogenase activity (S_DH_)

The ICM_6_ had the highest S_DH_ which was similar with the ICM_7&8_, but significantly greater than the ICM_1-5_ to the tune of 7.8–21% in the top 0.00–0.05 m soil depth. Further, in the second depth (0.05–0.15 m), the ICM_8_ recorded the highest S_DH_, wherein it was similar to the ICM_6&7,_ but shown 17–36.6% greater S_DH_ than the ICM_1-5_. In the 0.15–0.30 m soil depth, ICM_5_ resulted in the highest S_DH_. Averaged across the soil depths, the ICM_6-8_ gave 4–21% higher S_DH_ than the ICM_1-5_ (Table 3).

### Soil alkaline phosphatase (S_AP_)

The highest S_AP_ in the top 0.00–0.05 m soil depth was recorded with the ICM_8_, being significantly higher than the ICM_1-5,_ but similar to the ICM_6&7_. Indeed, the ICM_7&8_ resulted in 8.3–32.3% higher S_AP_ compared to the ICM_1-5_. While, in the 0.05–0.15 m soil depth, the highest S_AP_ was observed with the ICM_6_, where it was significantly more than the ICM_1-4&8_, but at par with the ICM_5&7_. Further, at 0.15–0.30 m, no significant difference in S_AP_ was noticed among the ICM modules (Table 3).

### Soil urease (U_RE_)

The U_RE_ in the 0.00–0.05 m soil depth was the highest with the ICM_8_, in which it was similar to the ICM_4-7_, but significantly greater than the ICM_1-3_. The increment in U_RE_ under ICM_7&8_ over the ICM_1-4_ (CT modules) was to the tune of 12.7–27.2%. Similarly, in the 0.05–0.15 m, the highest U_RE_ was recorded with the ICM_8_, which was significantly greater than the ICM_1-4,_ but similar to the ICM_5-7_. As expected, the ICM_7&8_ produced 8–27% higher U_RE_ compared to the ICM_1-4_. However, in the lowest soil layer (0.15–0.30 m), no significant differences in U_RE_ were observed among the ICM modules (Table 3).

### Sustainable yield index (S_YI_)

**Figure 5:**
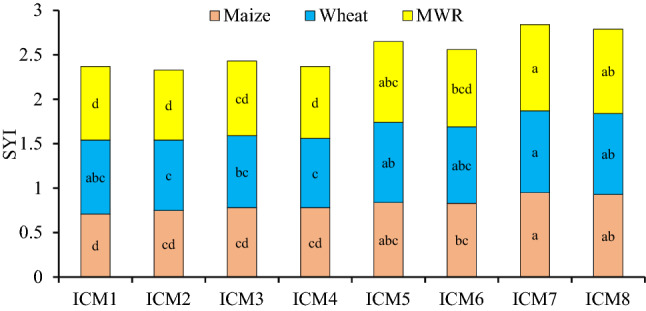
Effect of ICM modules on sustainable yield index (S_YI_) of the maize, wheat and maize-wheat rotation. Means followed by a similar lowercase letter within a bar are not significantly different at p < 0.05 using Tukey’s HSD test.

Among the ICM modules in the maize, the ICM_7_ had the greater S_YI_, but being at par to the ICM_1,5,6&8_, which was 12–15.2% greater than the ICM_2-4_. Again, S_YI_ in wheat was the highest under the ICM_7_, similar with ICM_5&8_, being 17.9–25.3% greater than the CT-based ICM_1-4_ modules. In the case of M_WR_, the S_YI_ was the highest under the ICM_7&8_, which was 13.4–18.6% higher than the ICM_1-4_, and similar to ICM_5_ (Fig. 5).

## Discussion

The rice–wheat is the commanding rotation in northern India’s ecologies. However, of late, from the resource exploitation to their judicious use for sustained yield, save water and improve soil-based properties is the focus^19,26^, besides achieving SDGs^12^. Seeing the degradation of natural resources, stagnation in crop yields and other constraints in adoption of rice–wheat rotation (R_WR_), it is thus noteworthy to identify the alternative crops and cropping rotations to sustain the food security. Maize ˈ*Queen of cereals*ˈ being a C_4_ plant, has wider adaptability under the diverse climate, thus could be a striking substitute of rice^2^. Every year, in the rice–wheat belt of north western India, the ground water falls off by 0.30–0.40 m^27^, and therefore, acreage under maize is likely to increase with the time. It is clearly evident that rice is the main water consumer^28^, maize could be a potential choice for accompanying wheat in this area, as it saves irrigation water, fulfils demand for palatable fodder and industries. Rice residue burning rather than returning to the soil, is another concern which not only deteriorates the air quality, but also have acute effects on human health^8^. Thus, the M_WR_ has a potential to replace the water guzzling rice under the R_WR_. The CA-based ICM practices in M_WR_ would intend for sustainable residue recycling, improve soil properties^19,29^ and sustain long-term production^30^.

Our findings confirmed the yield gains (14.6%, maize; 11.2%, wheat) under the CA-based ICM_5-8_ over the ICM_1-4_, however, the M_GEY_ enhanced by 12.3% (5 years’ av.). The ICM_5-8_, proved superior because of ZT, crops residue, and eventually the efficient use of inputs^31,32^ along with L_BFs_ consortia and A_MF_. Most soil organic matter (S_OM_) originates from the residues, and crops produce is positively linked with S_OM_^33^; crops residue retention helps S_OM_ build up, soil temperature moderation, improved water holding capacity, microbial and enzymatic activities, and nutrients mobilization in the rhizospheric zone^34,35^. In cereals, A_MF_ has extraordinary importance in boosting the yields^36^, and has capacity to acquire immobile nutrients beyond the radius of roots through their hyphal network^37,38^ owing to greater nutrients/water taken up^39,40^, ultimately improve yields^41,42^.

**Figure 6:**
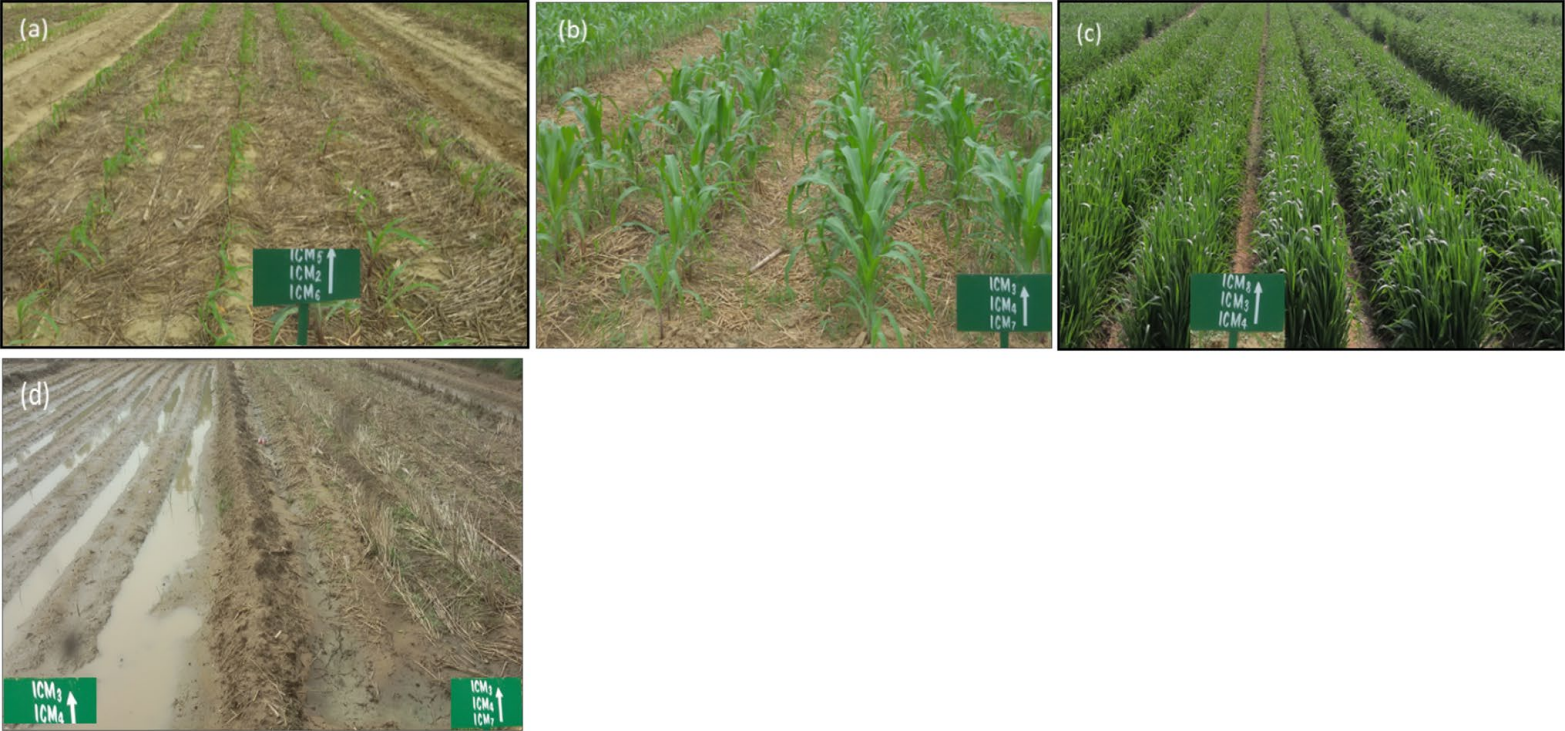
Initial establishment of ZT maize under residue retained CA-based ICM_6_ (**a**); 27 d old maize under CA-based ICM_7_ (**b**); raised bed wheat in ICM_4_ (**c**); soil conditions of CT-based ICM_4_ (water stagnation, left side) and CA-based residue retained ICM_7_ (no water stagnation, right side), photo clicked after 4–5 h of rain (**d**).

ICM_5-8_ increased 0.49 Mg ha^−1^ pooled wheat yield, but was 0.73 Mg ha^−1^ in maize, whereas, the yield advantage was more (0.96 Mg ha^−1^) with ZT bed planted maize (ICM_7-8_) than to the ICM_1-4_ (Table 1, Fig. 1). Excess (heavy rains) and deficit (longer dry spells) moisture are the common obstacles in the rainy season maize ecologies, but such variability does not exist during winters (wheat season). Residue retention in the ICM_5-8_ infiltrate more water (Fig. 6d), and creates better aeration for the maize crop, bed planted maize (ICM_7&8_) combining residues recorded yield advantage. Some meta-analysis studies have shown that the A_MF_ helps to tolerate such stresses^43,44^. The L_BFs_ fixes atmospheric-N and helps in solubilizing the insoluble P compounds which facilitate nutrient uptake, and improves the soil fertility, thereby, reduces the rate of chemical fertilizers up to 25%.

Water productivity (W_P_) is the crop yield unit^−1^ of water consumption. Five years’ results delineated that the ICM_5-8_ could save ~ 7% irrigation water, compared to the ICM_1&2_ (Fig. 2a). Long-time ZT tilled conditions where residues are retained, not only conserve the soil water, but facilitate better moisture regimes in the effective rhizosphere, and resulted in greater W_P_^32,45^. In the ICM_5-8_ modules, the surface residues could reduce the losses of water vapour and retained moisture for the longer period, thus requiring lesser irrigations. Further, the bed planting coupled with the crops residues has twin benefits of greater infiltration and lower water application rates^4,46,47,80^. In 2017–18 and 2018–19, the higher W_P_ was associated with the least water input coupled with greater yields than in other years (Fig. 2b).

Modules ICM_5-6_ being lesser expensive, on account of lesser tillage operations involved and thus saved labor costs in various physical field operations, whereas, the ICM_7-8_ were relatively costlier as these involved extra expenses in reshaping the beds (Fig. 3a). While, the ICM_1&4_ incurred the highest cost owing to more trafficking in different tillage operations^48^. The sequential tillage included the extra fuel cost, eventually these modules gave lower yield, as indicated in the inclination of economic net returns^5^. Of course, the timely sowing of the succeeding wheat under the ZT conditions gave yield advantage^49,50^ with the improved economic returns^48^. These results also reinforce the earlier research work in the adjoining ecologies^32,49,51^.

The ICM based agronomic management have vital role in the soil profile activities, and sustaining the soil health in the long-run^52^. Continuous crop residues recycling significantly improves the S_OC_ fractions^53^ and total S_OC_^45^. These CA-based practices have been widely analyzed for improving the S_OC_ and the microbial population size^54^. Interestingly, over the years, the ZT + residues could increase the S_OC_, particularly by releasing the considerable rhizo-depositions through hidden half and lower decaying rates^40^. Our results showed that the S_OC_ changed remarkably in the top soil layers, and ICM_5-8_ increased the S_OC_ storage by 12.1% in the top soil layer over the CT-based ICM_1-4_ (Fig. 4a), as intensive tillage operations facilitate the loss of S_OC,_ which is undesirable for the global C balance^45,55^.

The S_MBC_ is the living component (i.e., bacteria and fungi) of S_OM_, being the key indicator for S_OC_. In spite of small size, being a labile pool of S_OM_^56^, it contributes to the transformation or cycling of S_OM_^57,58^. In this study, the CA-based residue retained modules had 13.7% greater S_MBC_ in the 0.0–0.15 soil layers than the modules where residues were removed (Fig. 4b), as regular residue addition accumulated the soil C that enhanced the S_MBC_ and other microbial activities^46,59^. Moreover, the ZT conditions with sufficient crops residue are more conducive for the fungal hyphae growth, with additional supply of A_MF_ along with L_BFs_ further enhanced the fungal population and diversity, which could play an important role in the C / N cycling through their hyphal networks^60^. The S_DH_ is the most intuitive bioindicators, describing the soil fertility^61^. It is associated with the S_OM_ oxidation, and its activity depends on the microorganisms’ abundance and activity^62^. Current results showed a 10.1% improvement in the S_DH_ activity under the CA-based ICM_5-8_ modules, over CT-based practices (Table 3). The S_MBC_ and S_DH_ activities are directly associated with the recycling of the organic amendments, such as, the crops residues^46,63^.

Phosphatase activity is needed for P-mineralization and release of the PO_4_^3−^ for the plant uptake. Often it is stated that the phosphatase activities (alkaline / acid) are greater in the P deficient soils^65^, and the current study soils are alkaline in nature (pH 7.9) with only 13 kg ha^−1^ available P. The P deficiency, residue addition and stoichiometric changes^66^ would exhilarate the phosphatase activity under the CA-based modules. The urease activity responsible for the N mineralization and NH_3_ release through hydrolysing the C–N bond of the amides^67^. The residue based ICMs recorded greater urease, as residues acts as a substrate for the urease, and eventually help in increasing the N availability for plant uptake. The S_OC_, S_MBC_, S_DH_, A_PA_ and U_RE_ activities are directly linked with and the soil biological properties, and hence the soil fertility. We conclude that the CA-based residue retained modules of M_WR_ improved crops yields, farm economic profitability, and conserved the soil moisture. Such practices could also supplement the nutrients, sustain the crop yields, conserve natural resources, especially water and boost up the soil microbial functions for the long-term sustainability.

## Conclusions

The five years' results clearly indicated the superiority of the CA-based residue retained ICM_5-8_ modules, which produced 9.5–14.3% greater system maize grain equivalents (M_GEY_) over the CT-based modules (ICM_1-4_). Further, the ICM_2-8_ saved 6.5–8.0% irrigation water, and ICM_5-8_ recorded 10.3–17.8% higher system W_P_ than the residue removed (ICM_1-4_) modules. Of course, the conventional modules (ICM_1-4_) were expensive, however, ICM_5-8_ gave 24.3–27.4% extra returns than the ICM_1-4_, eventually made them economically more profitable. The residue retained modules (ICM_5-8_) registered 7.1–14.3% (0.0–0.15 m) greater S_OC_ than the ICM_1-4_, indicating the positive impacts of the residue addition which would be useful in sustaining the soil health in long-run. On an average, in 0.0–0.15 m depths, the soil biological activities i.e., S_MBC_ (10.1–16.7%), S_DH_ (10–15.6%), S_AP_ (14.8–18.1%), and U_RE_ (16.5–20%) increased in the ICM_5-8_ compared to the ICM_1-4_, thus the effect of residue retention was more pronounced in the upper soil layers than in lower depths. Further, there is a need to change regional growers’ perceptions towards the adoption of maize, it could be a potential choice for accompanying wheat in this area. Therefore, the ZT residue retained modules either ICM_7&8_ or ICM_5&6_ could be acceptable for their adoption in the M_WR_ for improving the yields, economic profitability and soil biological properties in the north western India and probably in other similar agro-ecologies.

## Materials and methods

### Experimental site, location and climate

Five years’ field experimentation on ICM was started in 2014–15 at the ICAR-Indian Agricultural Research Institute (28°35′ N latitude, 77°12′ E longitude, 229 m MSL), New Delhi, India. The study site comes under the 'Trans I_GPs_', being semi-arid with an average annual rainfall of 650 mm, of which ~ 80% occurs in July–September (south-west monsoon). The mean max. / min. air temperature ranges between 20-40ºC and 4-28ºC, respectively. The five years (2014–2019) weather data were recorded from the observatory adjoining to the experimental field, and presented in Supplementary Table 1. Before start of the experiment, a rainy season *Sesbania* was grown in 2014 to ensure the uniform fertility across the blocks. Initial soil samples (0.0–0.15 m depth) were collected in October 2014 after incorporating the *Sesbania* residues in soil. The soil samples were processed for the chemical analysis. The study site had a pH of 7.9 (1:2.5 soil and water ratio)^68^, 3.8 g kg^−1^ soil organic-C^69^, 94.1 kg ha^−1^ KMnO_4_ oxidizable N^70^, 97 µg g^−1^ soil microbial biomass carbon^71^, 51.3 μg PNP g^−1^ soil h^−1^ alkaline phosphatase^72^, 53.0 μg TPF g^−1^ soil d^−1^ dehydrogenase^73^, and 13.5 μg NH_4_-N g^−1^ soil h^−1^urease^74^.

### Description of different ICM modules

The eight ICM modules were tested, comprising of four conventional tillage (CT)-based (ICM_1-4_) and four conservation agriculture (CA)-based (ICM_5-8_) modules, replicated thrice in a complete randomized block design with the plot size of 60 m^2^ (15 m × 4.5 m) (Table 4). The crop residues were completely removed in the CT-based modules (ICM_1-4_), while in the ICM_5-8_ modules, *in-situ* wheat (~ 3 Mg ha^−1^ on dry weight basis)) and maize (~ 5 Mg ha^−1^, on dry weight basis) residues were retained on the soil surface during all the seasons of crops cultivation (Footnote Table 4, Fig. 6a,b).

**Table 4:** Description of integrated crop management (ICM) modules adopted in maize and wheat crops during the five yearsˈ fixed plot experimentation.

Treatment notations	Maize	Wheat
ICM_1_	CT_FB_ + 100% R_DF_	CT_FB_ + 100% R_DF_
ICM_2_	CT_FB_ + 75% R_DF_ + A_MF_ + L_BFs_	CT_FB_ + 75% R_DF_ + A_MF_ + L_BFs_
ICM_3_	CT_RB_ + 100% R_DF_	CT_RB_ + 100% R_DF_
ICM_4_	CT_RB_ + 75% R_DF_ + A_MF_ + L_BFs_	CT_RB_ + 75% R_DF_ + A_MF_ + L_BFs_
ICM_5_	ZT_M_ + W_R_ + 100% R_DF_	ZT_W_ + M_R_ + 100% R_DF_
ICM_6_	ZT_M_ + W_R_ + 75% R_DF_ + A_MF_ + L_BFs_	ZT_W_ + M_R_ + 75% R_DF_ + A_MF_ + L_BFs_
ICM_7_	ZT_RB_ + W_R_ + 100% R_DF_	ZT_RB_ + M_R_ + 100% R_DF_
ICM_8_	ZT_RB_ + W_R_ + 75% R_DF_ + A_MF_ + L_BFs_	ZT_RB_ + M_R_ + 75% R_DF_ + A_MF_ + L_BFs_

^#^ICM_1&2_ = conventional flatbed maize & wheat (CT_FB_); ICM_3&4_ = conventional raised bed maize & wheat (CT_RB_); ICM_5&6_ = zero-till (ZT) flatbed maize with wheat residue at ~ 3 Mg ha^−1^ (ZT_M_ + W_R_) & ZT wheat with maize residue at ~ 5 Mg ha^−1^ (ZT_W_ + M_R_), and ICM_7&8_ = ZT raised bed maize with wheat residue (ZT_RB_ + W_R_) & ZT wheat with maize residue (ZT_RB_ + M_R_). ^#^R_DF_ = recommended fertilizers for maize / wheat 150:26.2:50 / 120:26:33 NPK kg ha^−1^; L_BFs_ = NPK liquid bio-fertilizer; A_MF_ = arbuscular mycorrhizal fungi.

^#^Integrated weed management (maize): ICM_1-4_ = atrazine-pre-emergence (P_E_) *fb* 1hand weeding (H_W_) mulch; ICM_5-8_ = glyphosate-preplant (P_P_) + atrazine-P_E_ *fb* I H_W_ mulch. I_WM_ (wheat): ICM_1-4_ = sulfosulfuron 75 + metsulfuran-methyl (total)-P_oE_; ICM_5-8_ = glyphosate-P_P_ *fb* pendimethalin-P_E_ & total P^oE^. ^#^Need-based integrated pest management (I_PM_) and disease management (I_DM_) were followed in all the I_CM_ modules.

In the ICM_1-4_ modules, the field preparation was carried out by sequential tillage operations, such as, deep ploughing using the disc harrow, cultivator/rotavator twice (0.15–0.20 m), followed by levelling in each season. In the ICM_3-4_, the raised beds of 0.70 m bed width (bed top 0.40 m and furrow 0.30 m) were formed during each cropping cycle using the tractor mounted bed planter, and simultaneously wheat sowing was done (Fig. 6c). In the case of maize, ridges (0.67 m length) were prepared using the ridge maker. In the CA-based ICM_5-8_ modules, the tillage operations, such as, seed and fertilizer placement were restricted to the crop row-zone in maize and wheat both. In the ICM_7&8_, the permanent raised beds (0.67 m mid-furrow to mid-furrow, 0.37 m wide flat tops, and 0.15 m furrow depth), were prepared (Fig. 6d). However, these beds were reshaped using the disc coulter at the end of each cropping cycle without disturbing the surface residues. The sowing was accomplished using the raised bed multi-crop planter.

### Cultural operations and the fertilizer application

During every season, the maize (cv. PMH 1) was sown in the first week of July using 20 kg seed ha^−1^. The wheat (cv. HD 2967) crop was sown in the first fortnight of November using the seed-cum fertilizer drill (ICM_1-2_), bed planter (ICM_3-4_) and zero-till seed drill (ICM_5-8_) at 100 kg seed ha^−1^. The chemical fertilizers (N, P and K) were applied as per the modules described in the footnote of Table 4. At sowing, the full doses of phosphorous (P) and potassium (K) were applied using the di-ammonium phosphate (DAP) and muriate of potash (MOP), and the nitrogen (N) supplied through DAP. The remaining N was top-dressed through urea in two equal splits after the first irrigation and tasseling / silking stages in maize, and crown root initiation and tillering stages of wheat. In the modules receiving ¾ fertilizers (ICM_2,4,6,8_), the seeds were treated with the NPK liquid bio-fertilizer (L_BFs_) (diluted 250 ml formulation 2.5 L of water ha^−1^), and an arbuscular mycorrhiza (A_MF_) was broadcasted at 12 kg ha^−1^ as has been described by^75^. This L_BFs_ had the microbial consortia of N-fixer (*Azotobacter chroococcum*), P (*Pseudomonas*) and K (*Bacillus decolorationis*) solubilizers, procured from the commercial biofertilizer production unit of the Microbiology Division, ICAR-Indian Agricultural Research Institute, New Delhi (Patentee: ICAR, Govt. of India). Weeds were managed by integrating the pre- and post-emergence herbicides, and their combinations along with the hand weeding-mulching, as mentioned in the concerned modules (Footnote Table 4). However, in the CA-based modules (ICM_5-8_), the non-selective herbicide glyphosate (1 kg ha^−1^) was used 10 days before the sowing. The need-based integrated insect-pests and disease management practices were followed uniformly across the modules.

### Soil sampling and analysis

Before start of the experiment, the soil sampling was done from 0.0–0.15 m depth. Afterwards, five random samples from each module from 0.0–0.30 m soil depth were collected at the flowering stage of 5th season wheat. These samples were taken from the three soil depths (0.0 to 0.05, 0.05–0.15 and 0.150–0.30 m) using the core sampler. The ground, air-dried soil samples, passed through a 0.2 mm sieve were used for the determination of the Walkley and Black organic carbon (S_OC_), as described by^76^. For the soil biological properties, the soil samples were processed, and stored at 5ºC for 18–24 h, then analyzed the soil microbial biomass carbon (S_MBC_), dehydrogenase (S_DH_), alkaline phosphate (S_AP_) and the urease (U_RE_) activities.

### The soil microbial biomass carbon (S_MBC_)

The S_MBC_ was measured using the fumigation extraction method as proposed by^71^. The pre-weighed samples from the respective soil depths were fumigated with the ethanol-free chloroform for the 24 h. Separately, a non–fumigated set was also maintained. Further, 0.5 M K_2_SO_4_ (soil: extractant 1:4) was added, and kept on a reciprocal shaker for 30 min. and then filtered through a Whatman No. 42 filter paper. OC of the filtrate was measured through the dichromate digestion, followed by the back titration with 0.05 N ferrous ammonium sulphate. The S_MBC_ was then calculated using the equation:

$${\text{S}}_{{{\text{MBC}}}} = {\text{EC }} \times { 2}.{64}$$

where, EC = (C_org_ in fumigated soil – C_org_ in non-fumigated soil), and expressed in µg C g^−1^ soil.

### The dehydrogenase activity (S_DH_)

The S_DH_ activity (μg TPF g^−1^ soil d^−1^) was assessed using the method of^73^. The soil sample (~ 6 g) was saturated with 1.0 ml freshly prepared 3% triphenyltetrazolium chloride (TTC), and then incubated for 24 h under the dark. Later on, the methanol was added to stop the enzyme activity, and the absorbance of the filtered aliquot was read at 485 nm.

### The alkaline phosphatase activity (S_AP_)

The A_PA_ activity was estimated in 1.0 g soil saturated with 4 ml of the modified universal buffer (MUB) along with 1 ml of p-nitrophenol phosphate followed by incubation at 37 °C for 1 h. After incubation, 1 ml of 0.5 M CaCl_2_ and 4 mL of NaOH were added and the contents filtered through Whatman No. 1 filter paper. The amount of p-nitrophenol in the sample was determined at 400 nm^72^ and the enzyme activity was expressed as µg p-NP g^−1^ soil h^−1^.

### The urease activity

Urease activity was measured using 10 g soil suspended in 2.5 ml of urea solution (0.5%). After incubating for a day at 37 °C, 50 ml of 1 M KCl solution was added. This was kept on a shaker for 30 min and the aliquot was filtered through Whatman No. 1 filter paper. To the filtrate (10 ml), 5 ml of sodium salicylate and 2 ml of 0.1% sodium dichloro-isocyanide solution were added and the green color developed was measured at 690 nm^74^. These values are reported as µg NH_4_-N g^−1^ soil h^−1^.

### Water application and productivity

In experimental modules, water was given through the controlled border irrigation method. The current meter was fixed in the main lined rectangular channel, and the water velocity was measured. To get the flow discharge, then multiplied with area of cross section of the channel. The following formulae were used to calculate the applied irrigation water quantity and depth^3^:

$${\text{Irrigation water applied }}\left( {\text{L}} \right) \, = {\text{ F }} \times {\text{ t (i)}}$$

$${\text{Depth }}\left( {{\text{mm}}} \right) \, = {\text{ L}} \div {\text{A}}/{ 1}000$$

where, F is flow rate (m^3^ s^−1^), t is time (s) taken in each irrigation in each module and A is area (m^2^).

The effective precipitation (E_P_, difference between total rainfall and the actual evapotranspiration) was calculated, and then E_P_ was added to the irrigation water applied to calculate the total water applied in each module. Across the maize and wheat modules (ICM_1-8_), irrigations were given at the critical growth stages, such as, knee high and silking / tasseling (maize) and crown root formation, maximum tillering, flowering, heading / milking (wheat) stages, and after long dry spell (≥ 10-days).

On the basis of the soil water depletion pattern (at the depth of 0.60 m), in each season, 3–6 irrigations were given to maize, while wheat received 5–8 irrigations per season or crop including the pre-sowing irrigation. The rainfall data were obtained from the meteorological observatory located in the adjoining field. The water productivity (kg grains ha^−1^ mm^−1^ of water) was measured as per the equation given below:

$${\text{Water productivity }} = {\text{ economic yield }}\left( {{\text{kg ha}}^{{ - {1}}} } \right)/{\text{ total water applied }}\left( {{\text{mm}}} \right)$$

Additionally, the systems water productivity (S_WP_) was also estimated by adding the water productivity (W_P_) of both maize and wheat crops grown under the M_WR_.

### Yield measurements

In each season, the maize and wheat crops were harvested during the months of October and April, respectively, leaving 0.75 m border rows from all the corners of each module. The crops were harvested from the net sampling area (6 m × 3 m, 18 m^2^) located at the center of each plot. Maize crop was harvested manually and the wheat by using the plot combine harvester. All the harvested produce was sun dried before threshing and the grain and straw / stover yields were weighed separately. The stover/straw yields were measured by subtracting the grain weight from the total biomass. To compare the total (system) productivity of the different ICM modules, the system yield was computed, taking maize as the base crop, i.e., the maize equivalent yield (M_GEY_) using the equation^20^:

$${\text{M}}_{{{\text{GEY}}}} \left( {{\text{Mg ha}}^{{ - {1}}} } \right) \, = {\text{ Ym }} + \, \left\{ {\left( {{\text{Yw }} \times {\text{ Pw}}} \right) \, \div {\text{ Pm}}} \right\}$$

where, Y_m_ = maize grain yield (Mg ha^−1^), Y_w_ = wheat grain yield (Mg ha^−1^), P_m_ = price of maize grain (US$ Mg^−1^) and P_w_ = price of wheat grain (US$ Mg^−1^).

#### Farm economics

Under different ICM modules, the variable production costs and economic returns were worked out based on the prevailing market prices for the respective years. The production costs included the cost of various inputs, such as, rental value of land, seeds, pesticides, L_BFs_ / consortia, A_MF_, labor, and machinery; tillage / sowing operations, irrigation, mineral fertilizers, plant protection, harvesting, and threshing etc. The costs for the crops’ residues were also considered. The system total returns were computed by adding the economic worth of the individual crop, however, the net returns were the differences between the total returns to the variable production costs of the respective module. The Govt. of India’s minimum support prices (MSP) were considered for the conversion of grain yield to the economic returns (profits) during the respective years. Further, the system net returns (S_NR_) were worked out by summing the net income from both maize and the wheat in Indian rupees (INR), and then converted to the US$, based on the exchange rates for different years.

### Sustainable yield index (S_YI_)

^77,78^described the S_YI_ as a quantitative measure of the sustainability of agricultural rotation/practice. The sustainability could be interpreted using the standard deviation (σ) values, where the lower values of the σ indicate the greater sustainability and vice-versa. Total crop productivity of maize and wheat under the different ICM modules was computed based on the five years' mean yield data. S_YI_ was calculated using equation^78^.

$${\text{S}}_{{{\text{YI}}}} = \, \left( {{-}{\overline{\text{Y}}}_{{{\text{a }}{-}}} \sigma_{{\text{n}}} {-}_{{1}} } \right) \, /{\text{ Y}}^{{{-}{1}}}_{{\text{m}}}$$

where, –ȳ_a_ is the average yield of the crops across the years under the specific management practice, σ_n–1_ is the standard deviation and Y^–1^_ m_ is the maximum yield obtained under the set of an ICM module.

### Statistical analysis

The GLM procedure of the SAS 9.4 (SAS Institute, 2003, Cary, NC) was used for the statistical analysis of all the data obtained from different ICM modules to analyze the variance (ANOVA) under the randomized block design^79^. Tukey’s honest significant difference test was employed to compare the mean effect of the treatments at p = 0.05.

Authors have confirmed that all the plant studies were carried out in accordance with relevant national, international or institutional guidelines.

## Supplementary Information

Supplementary Information.
